# A human-specific allelic group of the *MHC DRB1* gene in primates

**DOI:** 10.1186/1880-6805-33-14

**Published:** 2014-06-13

**Authors:** Yoshiki Yasukochi, Yoko Satta

**Affiliations:** 1 Molecular and Genetic Epidemiology, Faculty of Medicine, University of Tsukuba, 305-8575 Tsukuba, Ibaraki, Japan; 2 Department of Evolutionary Studies of Biosystems, The Graduate University for Advanced Studies (SOKENDAI), 240-0193 Hayama, Kanagawa, Japan

**Keywords:** Allelic lineage, Balancing selection, HLA, Out-of-Africa, Pathogen, Trans-species polymorphism

## Abstract

**Background:**

Diversity among human leukocyte antigen (HLA) molecules has been maintained by host-pathogen coevolution over a long period of time. Reflecting this diversity, the *HLA* loci are the most polymorphic in the human genome. One characteristic of *HLA* diversity is long-term persistence of allelic lineages, which causes trans-species polymorphisms to be shared among closely related species. Modern humans have disseminated across the world after their exodus from Africa, while chimpanzees have remained in Africa since the speciation event between humans and chimpanzees. It is thought that modern humans have recently acquired resistance to novel pathogens outside Africa. In the present study, we investigated *HLA* alleles that could contribute to this local adaptation in humans and also studied the contribution of natural selection to human evolution by using molecular data.

**Results:**

Phylogenetic analysis of *HLA-DRB1* genes identified two major groups, *HLA* Groups A and B. Group A formed a monophyletic clade distinct from *DRB1* alleles in other Catarrhini, suggesting that Group A is a human-specific allelic group. Our estimates of divergence time suggested that seven *HLA-DRB1* Group A allelic lineages in humans have been maintained since before the speciation event between humans and chimpanzees, while chimpanzees possess only one *DRB1* allelic lineage (Patr-DRB1*03), which is a sister group to Group A. Experimental data showed that some Group A alleles bound to peptides derived from human-specific pathogens. Of the Group A alleles, three exist at high frequencies in several local populations outside Africa.

**Conclusions:**

*HLA* Group A alleles are likely to have been retained in human lineages for a long period of time and have not expanded since the divergence of humans and chimpanzees. On the other hand, most orthologs of *HLA* Group A alleles may have been lost in the chimpanzee due to differences in selective pressures. The presence of alleles with high frequency outside of Africa suggests these HLA molecules result from the local adaptations of humans. Our study helps elucidate the mechanism by which the human adaptive immune system has coevolved with pathogens over a long period of time.

## Background

Modern humans (*Homo sapiens*) live in a wide variety of environments, ranging from polar to tropical regions. Physiological anthropologists have long addressed the issue of ‘human adaptation’ to a variety of environments (that is the ability of humans to survive in a changing environment). Molecular evolution and population genetics also focus on the adaptation of humans to environmental changes. The approach of physiological anthropology is mainly to investigate differences in physiological modifications among individuals or ethnic groups in various environments (‘physiological polymorphism’) in order to understand human adaptation. On the other hand, molecular evolution or population genetics seek indications of natural selection by comparing nucleotide sequences of a target gene. If a new mutation at a target locus confers advantage for fitness in a certain environment, such a mutation is expected to rapidly spread throughout a population because of positive natural selection. Methods to detect such a signal of natural selection have been developed. For instance, in a protein coding gene, an excess in the number of non-synonymous substitutions (that change the amino acid sequence) over synonymous substitutions (neutral mutation) suggests that positive selection or balancing selection has occurred during the evolution of the target gene. In addition, the relationship between an allelic frequency and the extent of linkage disequilibrium (LD) around the selected mutation help us to find an allele that has rapidly spread in a population [1]. The advantageous allele is expected to dramatically increase its frequency in a short time so that recombination does not substantially break down the LD around the selected site.

Humans live in various environments around the world. The endemic pathogens that humans are infected by in these areas differ and humans have evolved to deal with these pathogens. In the present study, we focus on polymorphisms in the major histocompatibility complex (MHC), which plays an important role in triggering immune reactions in response to pathogens, and we discuss the possibility that a human-specific MHC allele is involved in the immunological adaptation to a human-specific pathogen.

The MHC is a set of cell-surface molecules that are responsible for presenting antigens from pathogens to lymphocytes in jawed vertebrates. As such, it is an important genetic system for protection against infectious disease [2]. In humans, the MHC is termed human leukocyte antigen (HLA). The *HLA* genomic region is located on the short arm of chromosome 6 at 6p21.3, spanning approximately 4 Mbp and comprising 224 genes [3]. The region is classified into three subregions: class I, class II, and class III regions. Among HLA molecules, six class I and II molecules (HLA-A, B, and C of class I and HLA-DR, DQ, and DP of class II) are important for antigen presentation to T lymphocytes. Class I molecules mainly bind to peptides from cytosolic proteins and the HLA-peptide complex is recognized by CD8^+^ T cells. Class II molecules present extracellular antigens to CD4^+^ T cells. Class I molecules consist of two polypeptide chains, an α heavy chain encoded in the class I region, and a β_2_-microglobulin light chain encoded on chromosome 15. Class II molecules are composed of two polypeptide chains, α and β chains, encoded in the class II region. For instance, the *DRA* and *DRB1* genes in the class II region encode the α and β chains, respectively, of the DR molecule. A peptide-binding region (PBR) was characterized with crystallography by Bjorkman *et al*. [4] for class I HLA-A and by Brown *et al*. [5] for class II HLA-DR. Molecular evolutionary studies of this region have revealed an enhancement of non-synonymous substitutions in the PBR, suggesting that the PBR is a target for balancing selection, which is responsible for the maintenance of *HLA* polymorphisms [6-10].

Polymorphisms in *HLA* genes have three unique features: (1) a large number of alleles, (2) a high degree of heterozygosity, and (3) remarkably long persistence time of the allelic lineage. These features are maintained by balancing selection but not by an increased mutation rate [11,12].

The chimpanzee (*Pan troglodytes*) is the closest extant relative of humans. Interestingly, chimpanzees appear to have resistance to several pathogens to which humans are susceptible, including HIV type 1 and human hepatitis B virus [13]. This indicates that the two species differ in their immune responses to these pathogens, and that possibly the pathogen recognition repertoire for MHC is different between the two species. Chimpanzees share some class II *DRB1* allelic lineages with humans [14-16]. In humans, genetic variation and selective intensity on *DRB1* are the greatest in the class II genes [17]. In humans, there are 13 *DRB1* allelic lineages (HLA-DRB1*01, *03, *04, *07, *08, *09, *10, *11, *12, *13, *14, *15 and *16), while there are only four allelic lineages (Patr-DRB1*02, *03, *07 and *10) in chimpanzees [14-16].

Chimpanzees have stayed in Africa since their divergence from humans approximately six million years ago (MYA). On the other hand, modern humans have dispersed across the world from Africa from 100,000 to 50,000 years ago and have adapted to regions with various exogenous pathogens. This begs the question of how modern humans have acquired resistance to a variety of pathogens in different environments. Therefore, the present study investigated the evolution of *HLA-DRB1* alleles that confer resistance to novel pathogens in humans. For this purpose, we studied nucleotide sequences of *HLA* genes using the IMGT/HLA database (http://www.ebi.ac.uk/imgt/hla/, [18]).

## Materials and methods

Nucleotide sequences of humans, chimpanzees, rhesus monkeys (*Macaca mulatta*), and crab-eating macaques (*Macaca fascicularis*) were used for phylogenetic analyses. A dataset of human *DRB* allele sequences, including *DRB1* and other functional *DRB* (*DRB3*, *DRB4*, and *DRB5*), was obtained from the IMGT/HLA database. The dataset of non-human primate *DRB1* alleles was obtained from the IPD MHC NHP database (http://www.ebi.ac.uk/ipd/mhc/nhp/, [19]). In the database, there were many partial coding sequences (CDS) (mainly exon 2 sequences). Using incomplete sequences is likely to be misleading in analysis of the phylogenetic relationships among sequences; therefore, we performed phylogenetic analyses only for full-length *DRB1* CDS. Because only partial sequences were available, we also excluded sequence data of the gorilla (*Gorilla gorilla*) and orangutan (*Pongo pygmaeus*) from the present analysis. We used two *HLA-DQB1* alleles as outgroup sequences. Next, we removed sequences of potential recombinant alleles according to a method that assumes a binomial distribution of the ratio of substitutions in a particular region to that in the entire region [17,20-22]. For phylogenetic analyses, we used 104 complete CDS: 56 *HLA-DRB1*, 6 *HLA-DRB3*, 4 *HLA-DRB4*, 2 *HLA-DRB5*, 11 chimpanzee *Patr-DRB1*, 22 rhesus monkey *Mamu-DRB1*, and 3 crab-eating macaque *Mafa-DRB1* alleles.

Brown *et al*. [5] identified 24 amino acids in the PBR of *HLA*-*DRB1* genes. In addition to the defined PBR, we included three amino acid sites (positions of 57, 67, and 90; for a total of 27 amino acids), because Brown and collaborators have subsequently shown that the three sites are involved in the formation of peptide-binding grooves and peptide binding [23].

Multiple sequence alignment of nucleotide sequences and phylogenetic-tree construction were performed using the MEGA v5.10 software [24]. A maximum likelihood (ML) tree for the non-PBR region was constructed based on the Hasegawa-Kishino-Yano (HKY) substitution model [25] with the nearest-neighbor-interchange (NNI) ML heuristic search. The best-fit substitution model was estimated by MEGA. Bootstrap analysis was performed using 1,000 replications. The numbers of non-synonymous substitutions per non-synonymous site (*d*_N_) and synonymous substitutions per synonymous site (*d*_S_) were calculated using the modified Nei-Gojobori method [26] with a Jukes-Cantor correction [27]. The transition/transversion bias used in this calculation was estimated with the ML method in MEGA. The average divergence time of *DRB1* alleles was estimated by the average of all pairwise *d*_S_ values, and the time to the most recent common ancestor (TMRCA) of alleles was estimated from the maximum number of synonymous substitutions per site (*d*_Smax_). The divergence time was estimated by the following formula:

$$\mathrm{TMRCA}=d_{\mathrm{Smax}}/2\mu$$

where *μ* is the neutral substitution rate of 10^−9^ per site per year at the MHC loci [9]. Pathogens recognized by HLA-DRB1 molecules were examined using the Immune Epitope Database (IEDB) (http://www.immuneepitope.org, [28]). Information about *HLA-DRB1* allele frequency among different human populations was collected from the NCBI dbMHC database (http://www.ncbi.nlm.nih.gov/gv/mhc, [29]).

## Results and discussion

### Two phylogenetic groups of *HLA-DRB1* alleles and human-specific *HLA* Group A

**Figure 1:**
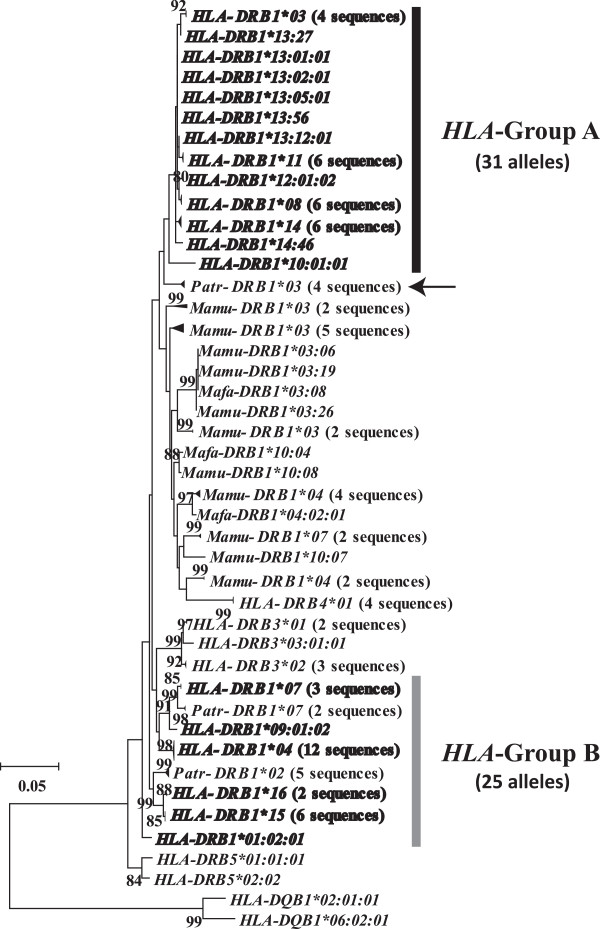
**Maximum likelihood tree for nucleotide sequences (690 bp) in the non-peptide-binding region (PBR) of *MHC DRB *alleles.** The sequence data of *MHC DRB* alleles, including those of humans, chimpanzees, and macaques, were obtained from IMGT/HLA and IPD databases. *HLA-DRB1* alleles are indicated in bold. Arrow indicates the Patr-DRB1*03 lineage, which is a sister group of *HLA* Group A alleles. Only bootstrap values > 80% are shown. Two *HLA-DQB1* sequences were used as an outgroup. The evolutionary distances were computed using the Hasegawa-Kishino-Yano (HKY) model. *HLA* Group A and *HLA* Group B indicate two major phylogenetic groups of *HLA-DRB1* alleles. *HLA*, humans; *Patr*, chimpanzees; *Mamu*, rhesus monkeys; *Mafa*, crab-eating macaques.

To examine phylogenetic relationships among *DRB* alleles in four primate species (*HLA-DRB1*/*3*/*4*/*5*, *Patr-DRB1*, *Mamu-DRB1*, and *Mafa-DRB1*), an ML tree was constructed from nucleotide sequences of the non-PBR region (Figure 1). Nucleotide sequences in the PBR were excluded for construction of the tree because they had an approximately ten-fold higher amino acid-altering (non-synonymous) substitution rate than synonymous substitutions due to balancing selection (Hughes and Nei [6,7]; Takahata and Nei [11]). When we focused on *HLA-DRB1* alleles, we identified two distinct clades in the ML tree. We refer to these two groups as *HLA* Group A and *HLA* Group B. Of the 13 known *HLA* allelic lineages, seven lineages, including DRB1*03, *08, *10, *11, *12, *13, and *14, were assigned to Group A, while the remaining six lineages, DRB1*01, *04, *07, *09, *15, and *16, were assigned to Group B.

In the ML tree, the Group B alleles showed trans-species evolution of polymorphisms with those in the chimpanzee (Patr-DRB1*02 and *07). Interestingly, 31 Group A alleles formed a monophyletic clade distinct from other primate *DRB1* alleles, although the bootstrap value for supporting this cluster was not particularly high, suggesting that the Group A alleles are human-specific. Previous studies [14-16] have not identified this *DRB1* monophyletic group in humans, because the nucleotide sequences used in those studies were limited to exon 2.

Both the mean and maximal *d*_S_ values were larger in Group B (mean *d*_S_, 0.041; *d*_Smax_, 0.082) than in Group A (mean *d*_S_, 0.018; *d*_Smax_, 0.057) (Table 1). This indicates that most allelic lineages in Group B have been maintained for a longer time than those in Group A. Additionally, Group A alleles may have diverged more recently than Group B alleles. Based on these results, we propose two hypotheses for the monophyly of Group A: (1) Group A alleles specifically expanded in the human lineage or (2) the orthologs to Group A alleles were lost in chimpanzees. We estimated the divergence time for alleles in each group in order to test these hypotheses.

**Table 1:** **The divergence time of the two *HLA *** **groups, *HLA*-Group A and *HLA*-Group B**

	***d***_**S **_**(mean)**	***T***	***d***_**S **_**(max)**	**TMRCA**
Group A	0.018	9 MYA	0.057	29 MYA
Group B	0.041	21 MYA	0.082	41 MYA

*T* indicates the mean divergence time of alleles. TMRCA indicates the time to the most recent common ancestor of alleles.

### Divergence time of alleles in *HLA* Groups A and B

The phylogeny showed a difference in divergence time between Groups A and B. The mean divergence times for Groups A and B were approximately 9 and 21 MYA, respectively, and TMRCAs were approximately 29 and 41 MYA, respectively (Table 1). These values suggest the presence of specific trans-species polymorphisms [10,30,31] in both groups, because the mean divergence time exceeded the speciation time of humans and chimpanzees [32-34]. Based on this result, we rejected the hypothesis that the *HLA* Group A allelic lineages specifically expanded in humans. However, the tree revealed that alleles in Group A did not intermingle with other non-human primate *DRB1* alleles (Figure 1). The closest was the Patr-DRB1*03 lineage cluster (indicated by an arrow in Figure 1).

**Figure 2:**
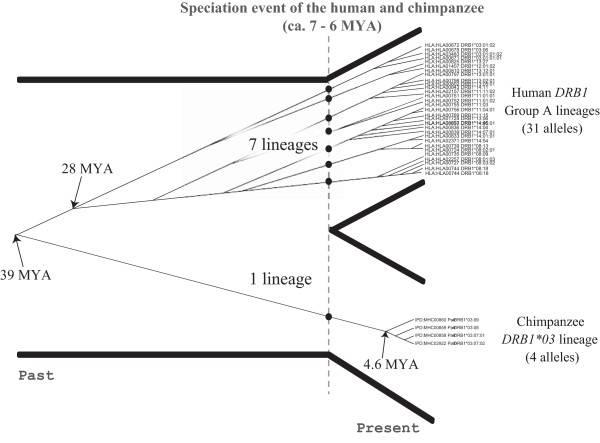
Divergence times of *HLA *Group A and Patr-DRB1*03 alleles. The dashed line represents the speciation event of humans and chimpanzees. Times to most recent common ancestor (TMRCAs) were estimated based on the maximum genetic distance at synonymous sites (*d*_Smax_).

Furthermore, we estimated the TMRCA of Patr-DRB1*03 cluster to be 4.6 MYA (Figure 2), suggesting that the alleles in this cluster diverged in chimpanzees after their divergence from humans. Accordingly, only one allelic lineage leading to the cluster in extant chimpanzees existed in the common ancestral population of humans and chimpanzees. On the other hand, in humans, pairwise *d*_S_ distances between *HLA-DRB1* alleles suggested that seven allelic lineages existed in the ancestral population (Figure 2). Therefore, the common ancestral population likely possessed at least eight allelic lineages.

Although alleles in Group A formed a single clade in the ML tree of primate *DRB* alleles, the TMRCA was 29 MYA, which is significantly older than six MYA (that is the speciation time of humans and chimpanzees). Thus, the molecular clock for *DRB1* alleles may have been skewed by various factors, such as back or parallel mutations (multiple mutations) or recombination/gene conversion. Indeed, in the Group A allele sequences, there was segregation of 21 synonymous sites. Among them, ten were singletons with a unique nucleotide observed only once in the sampled alleles, and 11 were phylogenetically informative sites. Among 55 pairs of 11 informative sites, 13 pairs were phylogenetically incompatible with each other. This incompatibility was likely the result of either recombination/gene conversion or multiple mutations at a single site. In the event of recombination/gene conversion, however, double recombination in a relatively small region or a conversion tract with a small size should be considered. Multiple mutations are a more likely cause of this incompatibility. To examine whether the presence of multiple substitutions masked an accurate estimate of the TMRCA, we tested the accuracy of the correction for multiple substitutions in the calculation of *d*_Smax_.

For this purpose, we estimated the maximum number of synonymous substitutions in a different way. First, we placed synonymous substitutions observed in the Group A alleles on each branch of the ML tree parsimoniously (Figure 1 and Additional file 1: Figure S1) and re-counted the number of synonymous substitutions (*K*_S_) in each pair of Group A alleles. The maximum *K*_S_ was thirteen (*K*_Smax_ = 13). TMRCA was calculated from this *K*_Smax_ divided by the mean number of synonymous sites (*L*_S_ = 223). As a result, the TMRCA of the Group A alleles was estimated to be 29 MYA. This showed good agreement with the TMRCA estimated by the Jukes-Cantor correction (29 MYA). Because there was no bias in our method of estimating TMRCA, we considered it to be reliable.

### Probability of maintaining seven human-specific *HLA* Group A allelic lineages over six million years

A method for calculating the probability, *g*_*nk*_(*t*) [35], that there were *k* allelic lineages among *n* extant lineages for *t* in *N* generations under balancing selection is available. In the present study, we tried to calculate the probability *g*_*nk*_(*t*) for seven ancestral allelic lineages being maintained since approximately six MYA among a sample of 31 Group A alleles (*n* = 31). However, because *HLA-DRB1* also contains the 25 Group B alleles, the 31 Group A sequences are only a part of the samples in the entire *HLA-DRB1.* There were no means to determine the effective population size (*N*_e_) of these subpopulations, which was required for the calculation of *g*_*nk*_(*t*); therefore, we could not calculate the probability of maintaining the current Group A alleles for six million years.

The effective population size *N*_e_ of modern humans is smaller than that of chimpanzees [36-38], and the eight allelic lineages in the ancestral population have likely been lost more frequently from the human lineage than the chimpanzee lineage. Nevertheless, the number of allelic lineages in humans is seven times larger than that in chimpanzees. This supports the hypothesis that natural selection selectively maintained Group A alleles in humans. It is important to understand the biological reasons why these seven lineages have been maintained only in humans.

### Specific peptides bound to *HLA* Group A alleles

It is possible that the *HLA* Group A allelic lineages have been because they bind to peptides derived from human-specific pathogens. Thus, we examined pathogens and their specific peptides recognized by each of the Group A and B allelic lineages based on information of experimental data from the IEDB database (Table 2). There were ten pathogens that produced peptides bound only by Group A alleles (for example, human papillomavirus type 11 (HPV-11) and influenza B virus (IBV)), and some of them were candidates for human-specific pathogens. In fact, in addition to HPV-11, *Bordetella pertussis* and measles viruses have been reported to be human-specific pathogens [39,40] (Table 2). Moreover, the IBV is restricted to humans with the exception of an infection identified in seals stranded on the Dutch coast [41]. At present, however, the repertoire of peptides bound by each allele is limited in the experimental data. Future studies will establish whether chimpanzees and macaques *MHC* are able to bind *HLA* Group A-specific peptides.

**Table 2:** The comparison of specific pathogen bound by HLA-DRB1 molecules between Group A and Group B

**Source organism ID**	**Source organism name**	**HLA-DRB1 molecule**	
520	*Bordetella pertussis*	HLA-DRB1*11:01	Group A
1309	*Streptococcus mutans*	HLA-DRB1*08:02	
6956	*Dermatophagoides pteronyssinus*	HLA-DRB1*11:01	
10408	Hepatitis B virus subtype adw2	HLA-DRB1*12:01	
10580	Human papillomavirus type 11	HLA-DRB1*03:01	
11234	Measles virus	HLA-DRB1*11:01, 11:03	
206203	Influenza B virus [B/Hong Kong/330/2001]	HLA-DRB1*14:01	
385630	Influenza A virus [A/Bangkok/1/1979(H3N2)]	HLA-DRB1*12:01	
387161	Influenza A virus [A/Japan/305/1957(H2N2)]	HLA-DRB1*11:01, 11:02, 11:03, 11:04	
10000974	*Streptococcus mutans* GS-5	HLA-DRB1*08:02	
210	*Helicobacter pylori*	HLA-DRB1*04:01, 15:01	Group B
236	*Brucella ovis*	HLA-DRB1*04:01, 04:07	
562	*Escherichia coli*	HLA-DRB1*04:01, 07:01, 15:01	
1313	*Streptococcus pneumoniae*	HLA-DRB1*15:01	
1358	*Lactococcus lactis*	HLA-DRB1*04:01, 04:07	
1423	*Bacillus subtilis*	HLA-DRB1*15:01	
1764	*Mycobacterium avium*	HLA-DRB1*15:01	
4479	Poaceae	HLA-DRB1*15:01	
10254	Vaccinia virus WR	HLA-DRB1*04:01	
10309	Herpes simplex virus (type 1/strain SC16)	HLA-DRB1*04:01, 15:01	
10957	Human rotavirus strain P	HLA-DRB1*04:01, 04:04, 15:01	
11309	Unidentified influenza virus	HLA-DRB1*04:01	
11679	HIV type 1 (CLONE 12)	HLA-DRB1*09:01	
11694	HIV virus type 1 (JH3 ISOLATE)	HLA-DRB1*09:01, 15:01	
12721	HIV	HLA-DRB1*01:03	
130763	Influenza A virus [A/Hong Kong/156/97(H5N1)]	HLA-DRB1*09:01	
197911	Influenza virus A	HLA-DRB1*04:01, 04:05	
211044	Influenza A virus [A/Puerto Rico/8/1934(H1N1)]	HLA-DRB1*04:01, 04:05	
243160	*Burkholderia mallei* ATCC 23344	HLA-DRB1*04:01	
351073	Mammalian orthoreovirus	HLA-DRB1*04:01, 15:01	
381512	Influenza A virus [A/New Caledonia/20/1999(H1N1)]	HLA-DRB1*04:01	
381513	Influenza A virus [A/Panama/2007/1999(H3N2)]	HLA-DRB1*04:01	
382781	Influenza A virus [A/Singapore/1/1957(H2N2)]	HLA-DRB1*09:01	
641501	Influenza A virus [A/California/04/2009(H1N1)]	HLA-DRB1*04:01	
746128	*Aspergillus fumigatus*	HLA-DRB1*15:01, 15:02	
768646	Influenza A virus [A/chicken/Uchal/8293/2006(H9N2)]	HLA-DRB1*09:01	
768647	Influenza A virus [A/chicken/Uchal/8286/2006(H9N2)]	HLA-DRB1*09:01	
991335	Influenza A virus [A/swine/Hong Kong/71/2009(H1N1)]	HLA-DRB1*09:01	

The dataset of source organism bound to HLA-DRB1 molecules was acquired from the MHC binding assay in the IEDB database.

In *HLA* Group B, although some pathogens infect not only humans but also other animals (for example, *Brucella ovis* and *Burkholderia mallei*), candidates for human-specific pathogens (for example, *Helicobacter pylori*) were included. This suggests that some Group B alleles might be also involved in local adaptation in humans.

The frequency distributions of eight *HLA-DRB1* alleles (HLA-DRB1*0301, *08:02, *11:01, *11:02, *11:03, *11:04, *12:01, and *14:01) that recognize Group A-specific pathogens were investigated using information in the NCBI dbMHC database (Additional file 2: Figure S2). The frequency distributions of HLA-DRB1*08:02, *12:01, and *14:01 were high outside Africa, suggesting that the frequency of the DRB1 molecules might have increased since the human species disseminated outside Africa.

Chimpanzees appear to have lost a relatively large number of alleles from the Group A allelic lineage while humans have maintained several allelic lineages since their speciation. The examination of genetic variation in MHC class I *Patr-A*, *Patr-B*, and *Patr-C* loci suggested that the genetic variations in chimpanzees have been severely reduced [42]. In this previous study, it was hypothesized that a selective sweep caused the loss of genetic diversity at MHC loci in chimpanzees in order to avoid widespread viral infection, such as that with chimpanzee-derived simian immunodeficiency virus, prior to a subspeciation of the common chimpanzee and bonobo (*Pan paniscus*) approximately two MYA. Although it is not known whether such selective sweep resulted in the loss of some *DRB1* allelic lineages in chimpanzees, reduced genetic variation at the three class I loci in chimpanzees may have been linked to the relatively small number of *Patr-DRB1* allelic lineages.

## Conclusions

A phylogenetic analysis of the *HLA-DRB1* gene identified two major groups of alleles, Groups A and B. Our findings suggest that Group A is human-specific and has been maintained by balancing selection in humans, while chimpanzees may have lost their counterparts to these allelic lineages due to different selective pressure. Some Group A alleles can bind to peptides derived from human specific pathogens and these showed a high frequency in populations outside Africa. Therefore, these alleles may have increased in frequency after the Out-of-Africa event. Our results imply that some of *HLA* Group A alleles may have contributed to local adaptation of humans.

## Perspective

In the present study, we identified a candidate human-specific *HLA-DRB1* allelic group. However, the sample size of chimpanzees was smaller than that of humans. Specifically, there were at least 88 chimpanzees used in published studies [14,15,43-45], while the *HLA-DRB1* alleles were detected in thousands of human individuals. Therefore, there is possible sampling bias among chimpanzees. The common chimpanzees are classified into at least four subspecies, which are, *Pan troglodytes troglodytes*, *P. t. verus*, *P. t. ellioti*, and *P. t. schweinfurthii*, in *Mammal Species of the World*[46]. In addition to the common chimpanzees, bonobo samples should also be included in the phylogenetic analyses of *DRB1* alleles. To exclude the possibility that our finding is an artifact of sampling bias, we plan to increase the sample size of chimpanzees in future studies, which will help validate the present estimates.

In the present study, *DRB1* alleles of rhesus monkeys and crab-eating macaques formed a taxon-specific clade with the exception of HLA-DRB4*01 sequences. All sampled alleles in the two macaques formed a sister clade with *HLA* Group A alleles in the ML tree but not with *HLA* Group B alleles (Figure 1). In the future, the reason why the *DRB1* alleles of macaques formed a large monophyletic group should be investigated.

It is difficult to verify that a molecule in *HLA* Group A can recognize human-specific pathogens. In recent years, there has been increasing information on peptide-*HLA* binding. Future studies must examine the relationships among *HLA* alleles, binding peptides, and pathogens in order to elucidate the mechanisms by which modern humans have adapted to a variety of environments around the world.

The contribution of natural selection to local adaptation in humans was evaluated from genomic data. The genomic data provide a universal framework for understanding human evolution and enable quantitative analysis of the operation of natural selection. We believe that molecular genetics techniques can shed light on some important issues in physiological anthropology.

## Abbreviations

bp: base pair; CDS: coding sequence; *d*_N_: number of non-synonymous substitutions per non-synonymous site; *d*_S_: number of synonymous substitutions per synonymous site; *d*_Smax_: maximum genetic distance at synonymous sites; HKY: Hasegawa-Kishino-Yano; HLA: human leukocyte antigen; HPV-11: human papillomavirus type 11; IBV: influenza B virus; IEDB: Immune Epitope Database; *K*_S_: number of synonymous substitutions; *K*_Smax_: maximum number of synonymous substitutions; LD: linkage disequilibrium; *L*_S_: mean number of synonymous site; MHC: major histocompatibility complex; ML: maximum likelihood; MYA: million years ago; *N*_e_: effective population size; NNI: nearest-neighbor-interchange; PBR: peptide-binding region; TMRCA: time to most recent common ancestor.

## Competing interests

The authors declare that they have no competing interests.

## Authors’ contributions

YY carried out data analysis, and wrote the draft of manuscript. YS was the primary supervisor. Both authors have read and approved the final manuscript.

## Supplementary Material

Additional file 1: Figure S1 Synonymous substitutions occurring on each branch of the ML tree for *HLA* Group A alleles in Figure 1. HLA-DRB1*09:01:02 was used as an outgroup sequence. The asterisk represents a synonymous substitution at a singleton. Click here for file

Additional file 2: Figure S2 Allelic frequency of HLA-DRB1 molecules that bind *HLA* Group A-specific pathogens. The allelic frequency data was obtained from the NCBI dbMHC database. Click here for file
