# Supplementary figures and images for: A human-specific allelic group of the MHC DRB1 gene in primates
Source: J Physiol Anthropol. 2014 Jun 13;33(1):14. doi: 10.1186/1880-6805-33-14 (PMC4072476; doi:10.1186/1880-6805-33-14)

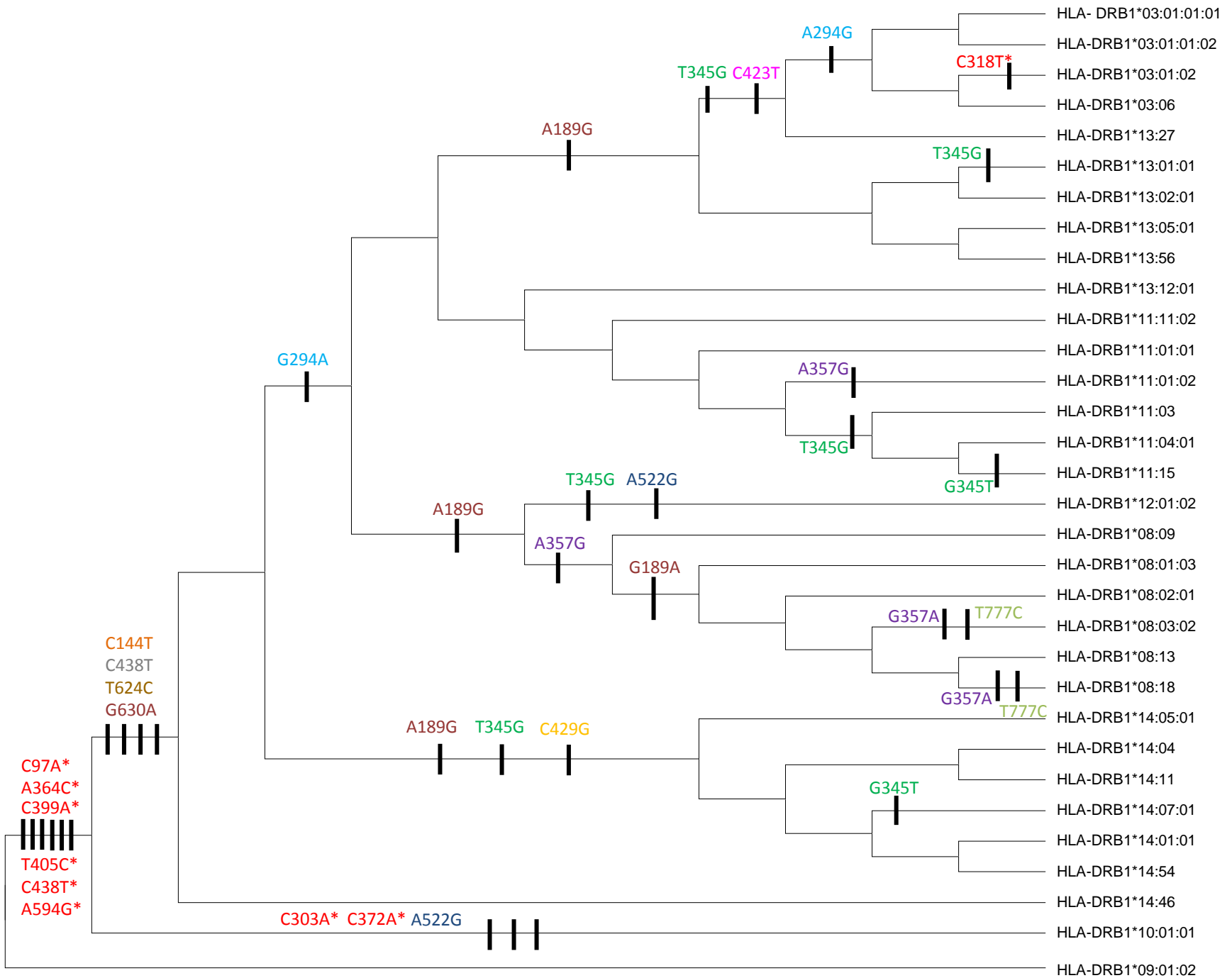

Supplement: Additional file 1: Figure S1 — Synonymous substitutions occurring on each branch of the ML tree for HLA Group A alleles in Figure 1. HLA-DRB1*09:01:02 was used as an outgroup sequence. The asterisk represents a synonymous substitution at a singleton. [file 1880-6805-33-14-S1.pdf]
