# Supplementary material for: A human-specific allelic group of the MHC DRB1 gene in primates
Source: J Physiol Anthropol. 2014 Jun 13;33(1):14. doi: 10.1186/1880-6805-33-14 (PMC4072476; doi:10.1186/1880-6805-33-14)
Supplement: Additional file 2: Figure S2 — Allelic frequency of HLA-DRB1 molecules that bind HLA Group A-specific pathogens. The allelic frequency data was obtained from the NCBI dbMHC database. [file 1880-6805-33-14-S2.pdf]

DRB1*0301

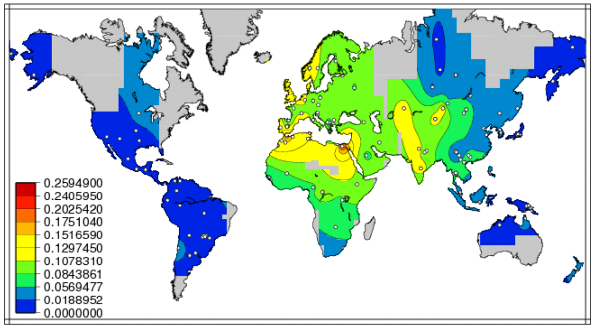

Image from Solberg et al. (2008) – see www.pypop.org/popdata for more info.

DRB1*0802

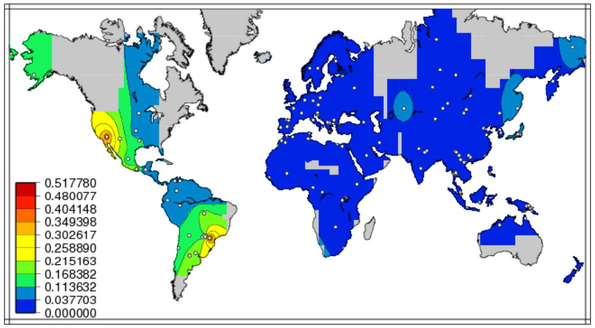

Image from Solberg et al. (2008) – see www.pypop.org/popdata for more info.

DRB1*1101

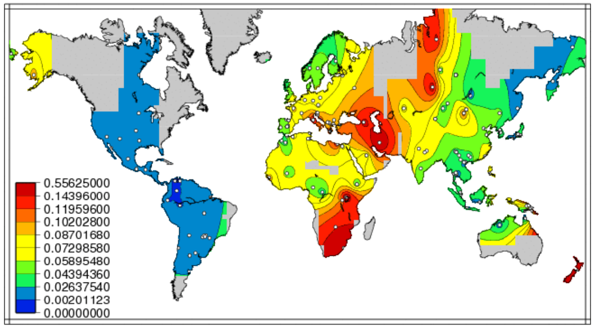

Image from Solberg et al. (2008) – see www.pypop.org/popdata for more info.

DRB1*1102

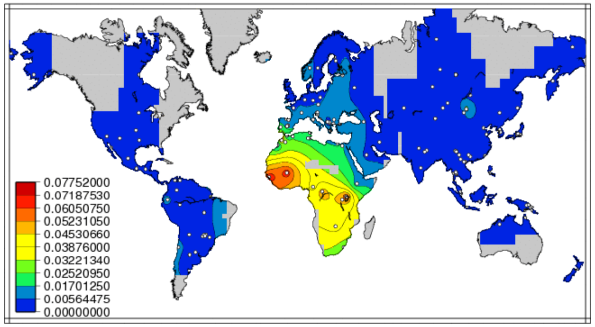

Image from Solberg et al. (2008) – see www.pypop.org/popdata for more info.

DRB1*1103

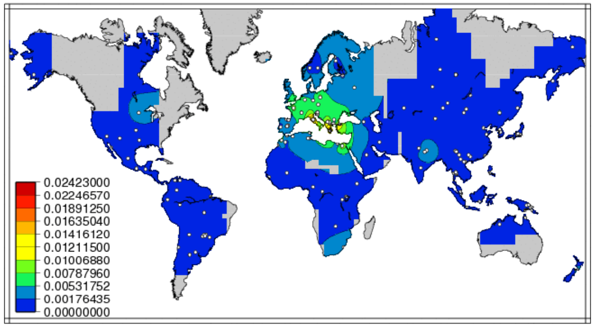

Image from Solberg et al. (2008) – see www.pypop.org/popdata for more info.

DRB1*1104

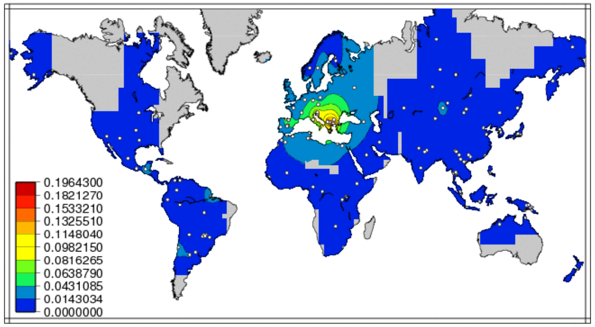

Image from Solberg et al. (2008) – see www.pypop.org/popdata for more info.

DRB1*1201

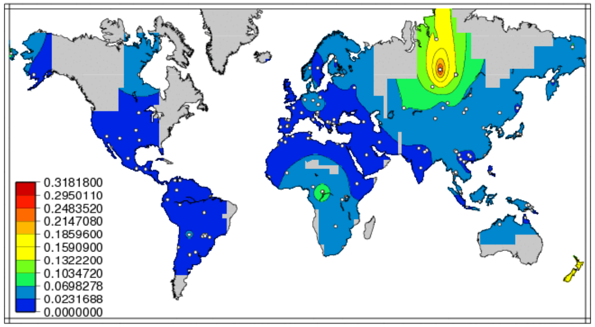

Image from Solberg et al. (2008) – see www.pypop.org/popdata for more info.

DRB1*1401

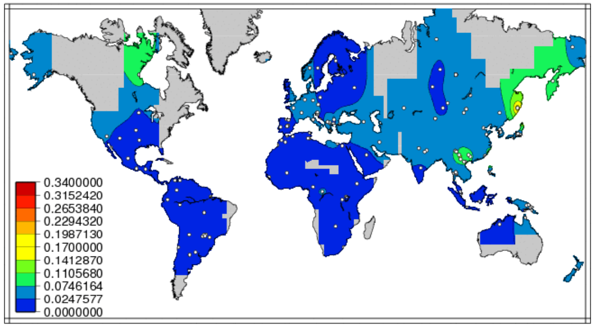

Image from Solberg et al. (2008) – see www.pypop.org/popdata for more info.

Allele frequency data in the NCBI dbMHC database (*http://www.ncbi.nlm.nih.gov/projects/gv/mhc/*)
